# RACIAL CLIMATE AND WILLINGNESS TO PARTICIPATE IN CLINICAL RESEARCH AMONG AFRICAN AMERICAN ADULTS

**DOI:** 10.1093/geroni/igae098.3700

**Published:** 2024-12-31

**Authors:** Sanaz Dabiri, Rema Raman, Jevay Grooms

**Affiliations:** University of Southern California, Arlington, Virginia, United States; University of Southern California, Los Angeles, California, United States; Howard University, Washington DC, District of Columbia, United States

## Abstract

In the US, there is a striking inequity in morbidity and mortality rates among racial and ethnic minoritized populations, particularly African American (AA) individuals, compared to White individuals. Despite these disparities, recruiting AA communities for clinical research remains challenging. Using the social-ecological model as a framework, this project aimed to examine whether the racial climate of the geographical area-including police shootings, neighborhood vulnerabilities, and individual trust in institutions- is significantly associated with willingness to participate in clinical studies. We analyzed data from the University of Florida HealthStreet registry, which assesses community perceptions toward research participation. This data was aligned with police shootings from the Fatal Encounters dataset and the Social Vulnerability Index by county and zip code. Participants had an average age of 42.6 years (SD=16.49) with an average education of 13.12 years (SD=2.5). Most were female (64%) and identified as AA (53.7%). Results showed that higher rates of police shootings were significantly associated with lower willingness to participate in studies involving blood samples (b=-1.39,se=.004, p<.01), taking medication (b=-.01,se=.006, p<.05), genetic studies (b=.001,se=.00, p<.05), and using medical equipment (b=-.02,se=.00,p<.001) among AA adults. Higher social vulnerability was also linked to lower research participation (b=-3.53,se=1.05, p<.001). Trust in research (ACME b=-.0004, p<.01) and researchers (ACME b=-.0003, p<.01) mediated the relationship between police shootings and willingness to take medication. Racial climate’s contribution to research participation among AA communities is poorly understood. Our findings underscore the need to address these factors to improve community engagement and reduce the diversity gap in research.

